# Primary mucosa-associated lymphoid tissue lymphoma in fallopian tubes: A case report and literature review

**DOI:** 10.1097/MD.0000000000030228

**Published:** 2022-08-26

**Authors:** Mengying Ji, Yichao Jin, Xing Chen, Yujing Li, Juveria Rahman, Huihua Dai

**Affiliations:** Department of Gynecology, the First Affiliated Hospital with Nanjing Medical University, Nanjing, China.

**Keywords:** cystic mass, fallopian tube, MALT lymphoma

## Abstract

Mucosa-associated lymphoid tissue (MALT) lymphoma accounts for 7%–8% of newly diagnosed lymphomas. The stomach is the most common organ of origin (nearly 50% of MALT lymphomas). Only 2% of MALT lymphomas arise from the female genital tract, and most originated from uterus. We described a rare case of MALT lymphoma primarily arising from the fallopian tube.

**Patient concerns:**

A 26-year-old woman was brought into our gynecological clinic because of a history of irregular menstruation. The transvaginal ultrasonography revealed cystic masses in bilateral adnexa and both of them showed rich vasculature. Laboratory tests showed a high level (455.3 U/mL) of cancer antigen-125 (CA-125).

**Interventions:**

A laparoscopy was performed.

**Outcomes:**

Frozen section of the removed specimen revealed acute and chronic inflammation with abundant inflammatory cells infiltrating the mesenchyme. The right fallopian tube was removed. However, the final histological results showed inflammation accompanied by hyperplasia of lymphoid tissue. Immunohistochemistry staining were consistent with MALT lymphoma. The patient received the second surgery to remove the left fallopian tube and also confirmed the same pathology.

**Lessons:**

Gynecologists should be aware of cystic masses which showed rich vasculature and high level of CA-125.

## 1. Introduction

Extranodal marginal zone B-cell lymphoma of mucosa-associated lymphoid tissue (MALT), also called MALT lymphoma is a subtype of non-Hodgkin lymphomas, which accounts for 7%–8% of newly diagnosed lymphomas.^[1]^ The stomach is the most common organ of origin (nearly 50% of MALT lymphomas). For extragastric organs of MALT lymphomas, ocular adnexa, salivary glands, thyroid gland, lung and skin are the most commonly involved.^[1]^ Only 2% of MALT lymphomas arise from the female genital tract, and most originated from uterus.^[2]^ We described a rare case of MALT lymphoma primarily arising from the fallopian tube.

## 2. Case presentation

A 26-year-old woman was brought into our gynecological department because of unexpected bilateral adnexal masses by ultrasound examination. The patient had been well until 2 months before this presentation, when she began to notice heavy menstrual bleeding. Transvaginal ultrasonography was performed and revealed a heterogeneous cyst measuring 38 × 18 × 42 mm beside the right ovary, and a complex cyst 37 × 30 × 36 mm in size adjacent to the left ovary. Both cysts showed rich vasculature. Laboratory studies of serum cancer antigen-125 (CA-125) showed a level of 455.3 U/ml (normal range: 0–35 U/mL). After a course of medical therapy, the ultrasound examination was performed again, which showed a fluid-filled tube with septations and kinking measuring 36 × 25 × 29 mm in size located adjacent to the right ovary, and a similar image was seen beside the left ovary measuring 27 × 16 × 21 mm. The level of serum CA-125 increased to 629.8 U/mL. Neither autoimmune disease nor other serious illness was detected. On pelvic examination, the vulva, vagina, and cervix were regular, the size of the uterus was average, and irregular cystic masses of about 3 cm diameter were palpated in the bilateral adnexa respectively.

**Figure 1.**
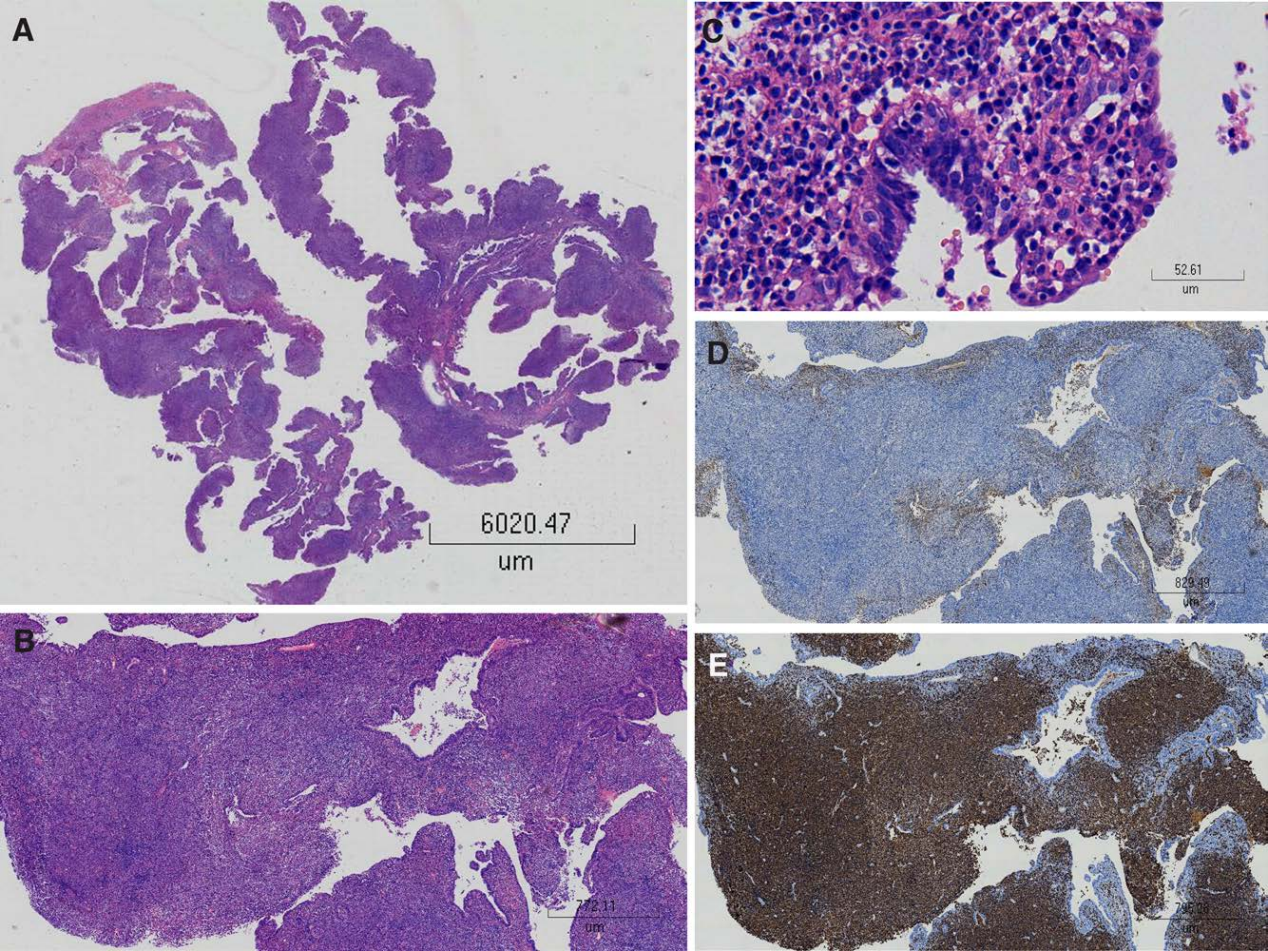
Pathological results of the removed fallopian tube. (A, B) Low-power field examination showed lymphoid cells diffusely infiltrated the whole oviduct layer that was destroyed, and only mucous epithelium remains. (C) High-power field examination showed lymphoid cells invaded the epithelium of the fallopian tube mucosa, the cells are small, and differentiated into mature lymphoid cells. (D) Kappa immunohistochemistry staining revealed the expression of Kappa was significantly reduced, suggesting that the lymphocytes were clonally expressed. (E) CD20 immunohistochemistry staining showed consistent B-lineage expression in the cells.

Exploratory laparoscopy was performed to determine whether the masses were malignant fallopian tube tumors or hydrosalpinges. It was found that the fimbriae of bilateral fallopian tubes were blocked, and the tubes were dilated about 3–3.5 cm in diameter, which adhered to the medial leaf of the broad ligament and ovaries. After detaching the adhesions and releasing the distortions, right and left salpingostomy and partial resection of fallopian tube fimbriae were conducted. Pus was found flowing from the left fallopian tube upon salpingostomy. Examination of the intraoperative frozen section of the removed specimen revealed acute and chronic inflammation with abundant inflammatory cells infiltrating the mesenchyme. However, after the surgery, the final histological results showed inflammation accompanied by hyperplasia of lymphoid tissue (Fig. 1). Immunohistochemistry staining revealed B-cell–associated antigens such as CD20 and CD79a were expressed, while CD3, CD5, CD23, and CD10 were negative (Fig. 1). The biopsy showed gene rearrangement of immunoglobulin heavy and light chain was positive. These findings were consistent with extranodal marginal zone B-cell lymphoma of MALT. After the surgery, a gene test of pathogenic microorganisms was also performed, revealing that no pathogen was found in the fallopian tube, such as bacteria, viruses, fungus, parasites, mycobacterium tuberculosis, mycoplasma, chlamydia, and rickettsia. As MALT lymphoma arising from the fallopian tube is rare, a multi-disciplinary team discussion was organized. Providing that MALT lymphoma was malignant and another tube may have been involved as seen during the operation, amounts of this extranodal MALT lymphoma might have been associated with specific infection; the result of multi-disciplinary team discussion suggested secondary surgery remove the left fallopian tube. The final pathology is consistent with MALT lymphoma.

According to the Ann Arbor staging system, the most widely used for MALT lymphomas, this case was diagnosed as stage I. Twelve months postoperative follow-up showed no tumor recurrence and no elevation in CA-125 levels.

## 3. Discussion

MALT lymphoma was first described in 1983, a subtype of non-Hodgkin lymphomas.^[1]^ Primarily, it was defined as a subclass of gastric lymphoma as the stomach was the most common site of the origin, but it has been reported that MALT lymphoma may arise in other organs, such as the ocular adnexa, salivary glands, thyroid gland, lung and skin.^[1]^ Even about 2% of MALT lymphomas arise from the female genital tract, most originated from uterus.^[2]^ In 1986, Morris and colleagues first reported mucosal-associated lymphoid tissue (MALT) in the fallopian tube.^[3]^ And so far, few case of MALT lymphoma primarily arising from the fallopian tube were reported.

It is well acknowledged that the pathogenesis of MALT lymphoma is associated with autoimmune disorders (such as Hashimoto thyroiditis, Sjögren syndrome) and infectious agents (such as *Helicobacter pylori* and *Campylobacter jejuni*).^[1]^ In previous cases of MALT lymphoma in the fallopian tube, just 1 case described Acinetobacter discovered in swab culture.^[4]^ In our case, no specific pathogen was found too in the gene test, proving that not all MALT lymphomas are caused by pathogen infection, especially in the fallopian tube. The relationships between these factors and the fallopian tube MALT lymphoma have not been determined yet.

Typically, patients with MALT lymphoma may present with fever, night sweats, and weight loss symptoms and have a history of chronic inflammation of the specific site or autoimmune disease. To our knowledge, when MALT lymphoma involving female genital tract, patients usually have a history of menorrhagia, dysmenorrhea, abdominal distention, bloating, or pelvic pain.^[2]^ According to the organ involved, the clinical manifestation of nongastric MALT lymphomas may be atypical and variable. Fallopian tube MALT lymphoma may mimic salpingitis, adnexal mass, or hydrosalpinx.^[5–7]^ Additionally, patients may not present with typical inflammation, infection, or autoimmune disorder signs. The levels of LDH and β_2_-microglobulin may also be typical.^[8]^ Due to this, it may be challenging to diagnose only depending on the patients’ presentation, history, or laboratory examination, and can be easily confused with gynecology malignant tumors. Interestingly, in this case, the patient presented with abnormal menstruation and high levels of CA-125, which was an indication for operation, after which the CA-125 level gradually decreased to the normal range.

After diagnosing MALT lymphoma, a comprehensive evaluation should be conducted, including Ann Arbor stage, nodal, and extranodal site involvement, and International Prognostic Index scores. These may be the prognostic factors of nongastric MALT lymphoma. In addition, the normal levels of serum LDH and a smaller number of giant cells may be associated with longer progression free survival.

MALT lymphoma has an indolent clinical course and a good prognosis, despite frequent relapses.^[9]^ Recurrences can occur in the same organ or other extranodal sites. In general, nongastric MALT lymphoma does not involve other lesions except the primary site. To date, no prognostic markers for the nongastric MALT lymphoma have been recognized, nor definite guidelines exist for its management. An appropriate individualized treatment plan should be provided depending on the clinicians’ experience. Surgery, radiotherapy and single-agent chemotherapy have shown efficacy in treating nongastric MALT lymphomas.^[10]^ But in any case, surgery should be considered first. Chemotherapy may be considered when the tumor is aggressive. Specific antibiotic treatment according to the sensitivity pattern is also necessary if a pathogen is found. Combined previous cases reported and the patient’s condition, Whether the surgical treatment was effective and successful is based on the combined postoperative situation and the 1-year follow-up. Attention should be paid to the postoperative lifelong clinical follow-up with ultrasonography and computed tomography scan of the chest and abdomen due to evidence of late relapses.

## 4. Conclusion

In conclusion, MALT lymphomas in the fallopian tubes are rare. Patients often do not have typical clinical presentation, and diagnosis depends on the final pathological findings. So it is difficult to be diagnosed early and can be easily confused with gynecology malignant tumors. It should be staged as soon as possible and requires targeted treatment. Pathogenesis of the disease and finding specific clinical manifestations can be the focus of research for future studies regarding nongastric MALT lymphomas.

## Acknowledgments

We thank pathologists of the First Affiliated Hospital with Nanjing Medical University to offer figures and diagnosis the disease.

## Author contributions

MJ and YJ wrote the article. XC and YL contributed to the data collection and analysis. XC, JR and HD contributed to the data interpretation and critical revision. All authors have read and approved the article.

Conceptualization: Huihua Dai.

Data curation: Xing Chen, Yujing Li.

Formal analysis: Xing Chen, Juveria Rahman.

Supervision: Xing Chen, Huihua Dai.

Writing—original draft: Mengying Ji, Yichao Jin.

Writing—review and editing: Xing Chen, Huihua Dai.
